# The BK channel interactome: molecular partnerships and biophysical implications

**DOI:** 10.1007/s12551-026-01432-z

**Published:** 2026-04-20

**Authors:** Nadya Yanes-Alonso, Bárbara Armas-Rijo, Andrea Reyes-Carrión, Rebeca Navarro-Alonso, Luis O. Romero, David Bartolomé-Martín, Teresa Giraldez

**Affiliations:** 1 Departamento de Ciencias Médicas Básicas, Facultad de Medicina, Universidad de La Laguna, San Cristóbal de La Laguna, Tenerife S38071 Spain; 2 Instituto de Tecnologías Biomédicas, Universidad de La Laguna, San Cristóbal de La Laguna, Tenerife S38071 Spain; 3 Departamento de Bioquímica, Microbiología, Biología Celular y Genética, Facultad de Ciencias, Universidad de La Laguna, San Cristóbal de La Laguna, Tenerife S38071 Spain

**Keywords:** Large-conductance Ca^2+^ and voltage-activated K^+^ channels (BK), Interactome, Protein-protein interactions, Multichannel complex

## Abstract

Large conductance voltage- and Ca^2^⁺-activated K⁺ (BK) channels are key regulators of membrane excitability and intracellular Ca^2^⁺ signaling in a wide range of excitable and non-excitable cells. Although the biophysical properties of BK channels have been extensively characterized, it has become increasingly clear that their functional diversity arises largely from interactions with a broad network of associated proteins. In this focused review, we provide an updated overview of the BK channel protein interactome, highlighting how these interactions shape channel gating, trafficking, localization, and coupling to Ca^2^⁺ sources. We discuss well-established regulatory partners, including β and γ auxiliary subunits, as well as emerging modulators such as BKIP-1 and LINGO-1. We further examine interactions with cytoskeletal and trafficking proteins that govern BK channel surface expression and mobility, and with Ca^2^⁺-permeable channels and receptors, including voltage-gated Ca^2^⁺ channels, TRP channels, ryanodine receptors, IP₃ receptors, and NMDA receptors, that create specialized Ca^2^⁺ signaling nanodomains. Finally, we summarize the roles of intracellular signaling molecules and scaffolding proteins that integrate BK channels into larger signaling assemblies. Together, these findings position BK channels as central hubs within multiprotein complexes that enable precise, context-dependent control of cellular excitability and Ca^2^⁺-dependent physiological processes.

## Introduction

Large conductance voltage- and Ca^2+^-activated K^+^ channels, commonly known as BK, MaxiK, Slo1, or KCa1.1 channels, were first identified more than 40 years ago as potassium currents modulated by both Ca^2+^ and voltage (Marty 1981; Pallotta et al. 1981). BK channels are typically found at the plasma membrane, where they assemble as homotetramers of α subunits encoded by the *KCNMA1* (*SLO1*) gene (Cui et al. 2009). This gene was originally cloned from *Drosophila* (Atkinson et al. 1991; Adelman et al. 1992) and later from mice (Butler et al. 1993) and humans (Dworetzky et al. 1994; McCobb et al. 1995). Notably, these channels are also functionally present in mitochondrial and nuclear membranes; however, the roles and regulatory mechanisms of these intracellular BK channels have been reviewed elsewhere (e.g., González-Sanabria et al. (2021)) and are not covered in detail in the present review, which focuses primarily on BK channel complexes associated with the plasma membrane. BK channels serve a wide range of specialized physiological functions across various excitable and non-excitable tissues, including smooth muscle (Dopico et al. 2018), kidney (Grimm and Sansom 2007), the gastrointestinal tract (Gonzalez-Perez et al. 2021), salivary glands (Yang et al. 2017), and bone (Henney et al. 2009). Within the central nervous system (CNS), they are highly expressed in somas, axons, dendrites, and synaptic terminals of neurons from the olfactory system, neocortex, basal ganglia, hippocampus, and thalamus (Sausbier et al. 2006; Wang et al. 2014; Trimmer 2015; Kshatri et al. 2018; Tazerart et al. 2022).

BK channels are activated through the synergistic interaction between intracellular Ca^2+^ and membrane depolarization (McManus and Magleby 1988; Rothberg and Magleby 1999; Horrigan and Aldrich 2002). Compared to other K^+^ channels, these channels show an exceptionally high conductance, ranging from 100 to 300 pS (Latorre et al. 2017). These unique properties make BK channels a crucial mediator between membrane excitability and intracellular Ca^2^⁺ signaling (Rothberg 2012). Accordingly, BK channel function has been involved in key physiological processes, such as action potential termination (Montgomery and Meredith 2012; Shah et al. 2021), neurotransmitter release (Robitaille et al. 1993; Lingle et al. 1996), plasticity and neurotransmission (Isaacson and Murphy 2001; Zhang et al. 2018a, b; Gomez et al. 2021), circadian rhythm regulation (Meredith et al. 2006), smooth muscle contraction (Nelson et al. 1995; Meredith et al. 2004), cardiac sinoatrial node firing (Lai et al. 2014), and insulin secretion (Houamed et al. 2010). Given their critical physiological roles, dysregulation of BK channels has been implicated in several genetic and acquired disorders (see recent reviews: Echeverria et al. (2024); Meredith (2024)).

**Fig. 1:**
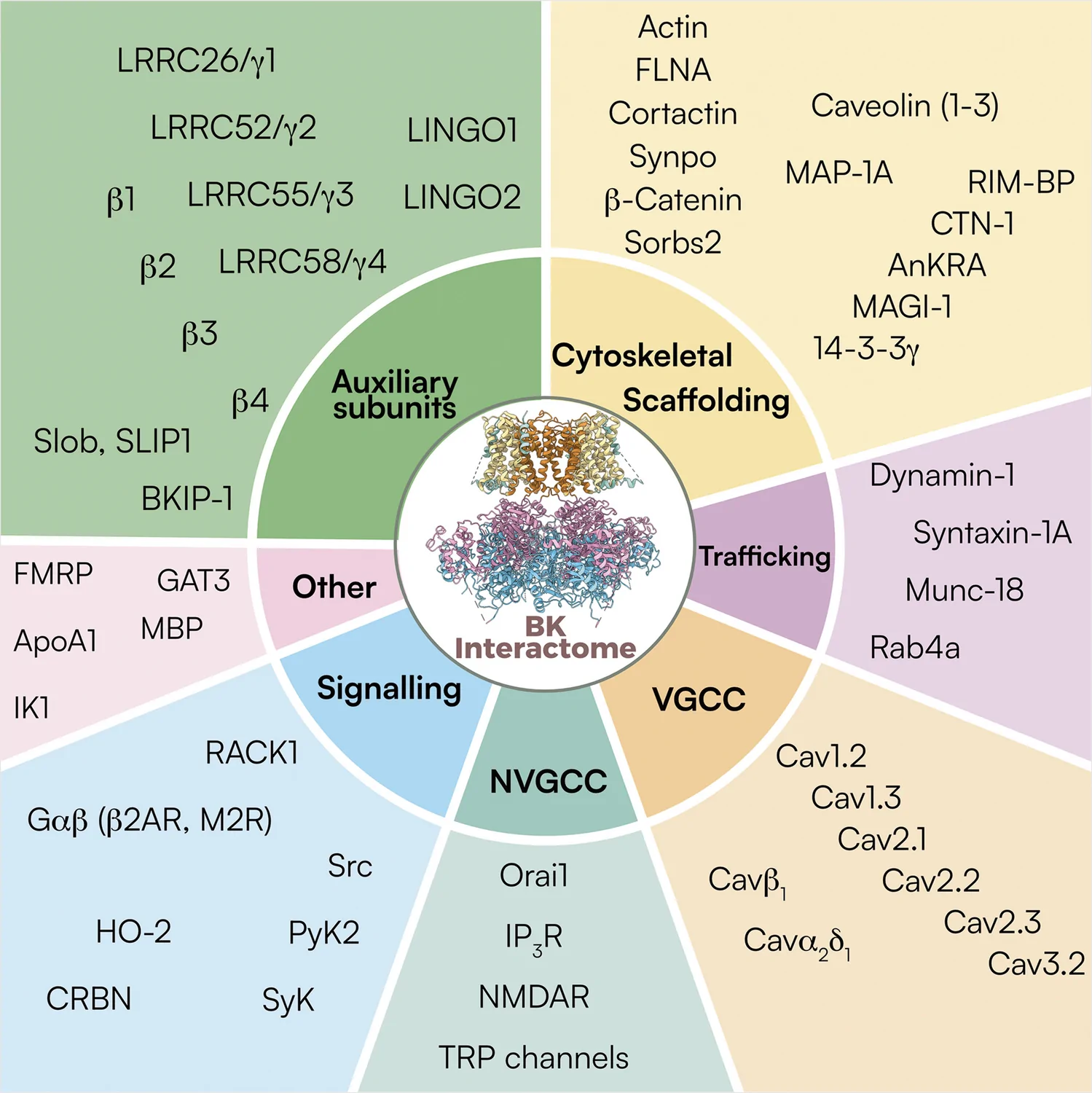
The BK channel interactome. Overview of proteins reported to interact with the large-conductance Ca^2^⁺- and voltage-activated K⁺ (BK, KCa1.1) channel. The BK channel α subunit structure is shown at the center, surrounded by functional classes of interacting proteins, grouped into BK channel auxiliary subunits, cytoskeletal/scaffolding proteins, trafficking proteins, Ca^2^⁺-permeant proteins (VGCC and NVGCC), intracellular signaling proteins, and other associated proteins. This organization highlights the diverse molecular partners that regulate BK channel localization, gating, signaling integration, and physiological function

The diverse physiological functions of the BK channel are driven largely by its interactions with a wide range of proteins with distinct roles. While its most well-characterized interactions involve regulatory subunits and Ca^2+^-permeant proteins, a large body of evidence demonstrates that BK also associates with proteins involved in intracellular signaling, protein trafficking, and other cellular functions. In this focused review, we provide an updated perspective on the BK protein interactome (see also a previous review on this topic by Kim and Oh (2016) and Pereira da Silva et al. (2022)), highlighting the biophysical and physiological significance of these interactions. Each section categorizes these interacting proteins into distinct families (regulatory subunits, cytoskeletal, scaffolding and trafficking proteins, Ca^2^⁺-permeable proteins, signaling molecules and membrane receptors, and other proteins that do not fit into these groups) with detailed information summarized in Table 1 and Fig. 1. Throughout this review, we primarily focus on proteins for which experimental evidence supports a direct physical interaction or complex formation with the BK channel. In a limited number of cases, proteins lacking definitive evidence for direct interaction are discussed when their proposed association with BK is strongly supported by functional data and is of clear biophysical or physiological relevance.

**Table 1:**
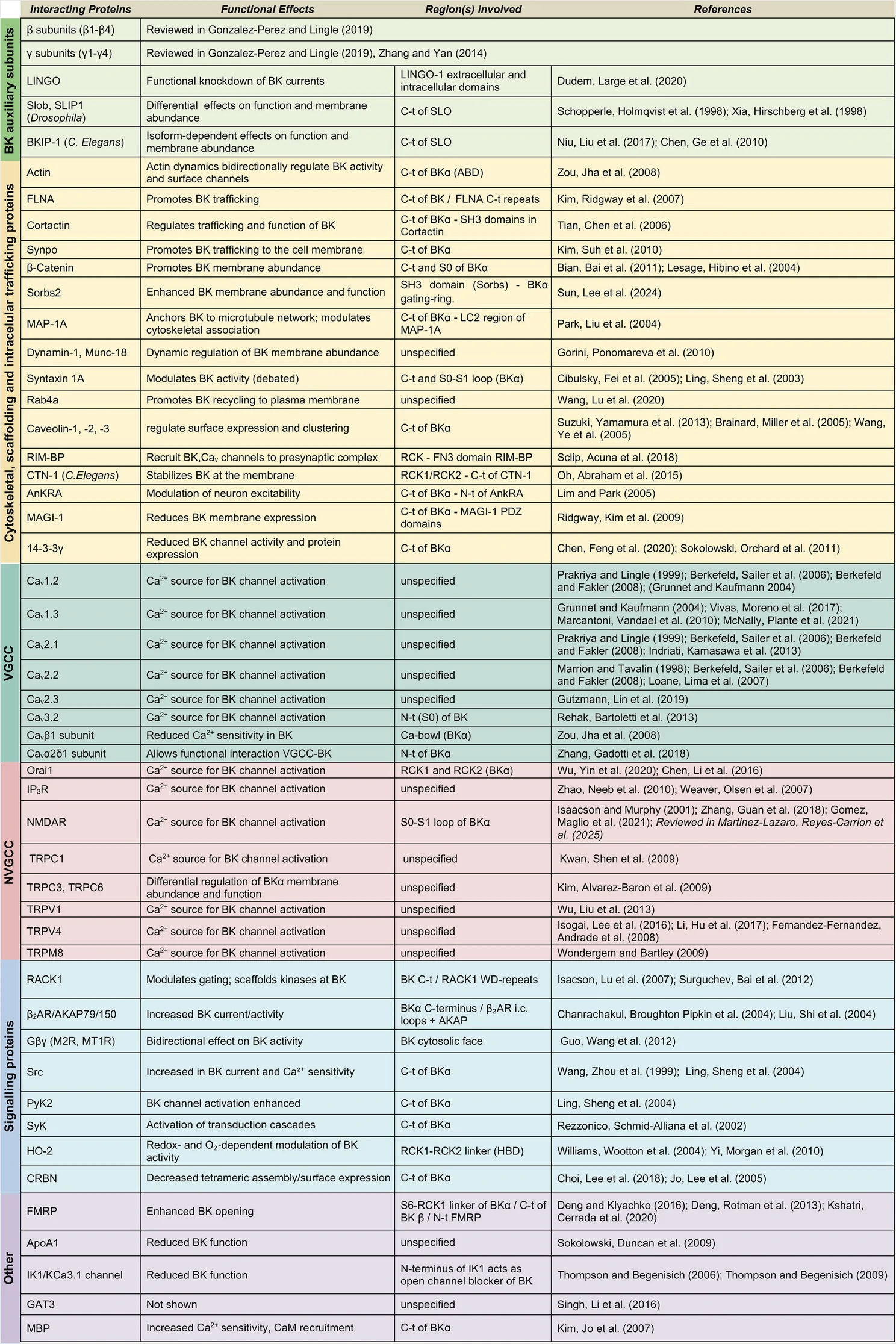
Summary of BK channel-interacting proteins reviewed in this work. Proteins are organized into functional categories corresponding to the sections in the main text. Inclusion in the table is based primarily on experimental evidence supporting physical association with BK channels within the context of plasma membrane signaling complexes. The channel regions listed correspond to the interaction sites reported in the primary literature; in several cases these are broadly defined (e.g., C-terminal region) due to limited structural mapping in the original studies. In a limited number of cases, proteins supported mainly by functional, biochemical, or cell type-specific evidence are listed; for these interactions, definitive structural interfaces remain to be established. Serine/threonine kinases (PKA, PKC, PKG, and CaMKII) are excluded from the table, as their effects on BK channels predominantly reflect phosphorylation-dependent regulation rather than stable physical interactions. Other proteins discussed in the text for which direct interaction has not been demonstrated are likewise excluded

*N-t* N-terminal domain, *C-t* C-terminal domain

## BK channels: functional and structural features

**Fig. 2:**
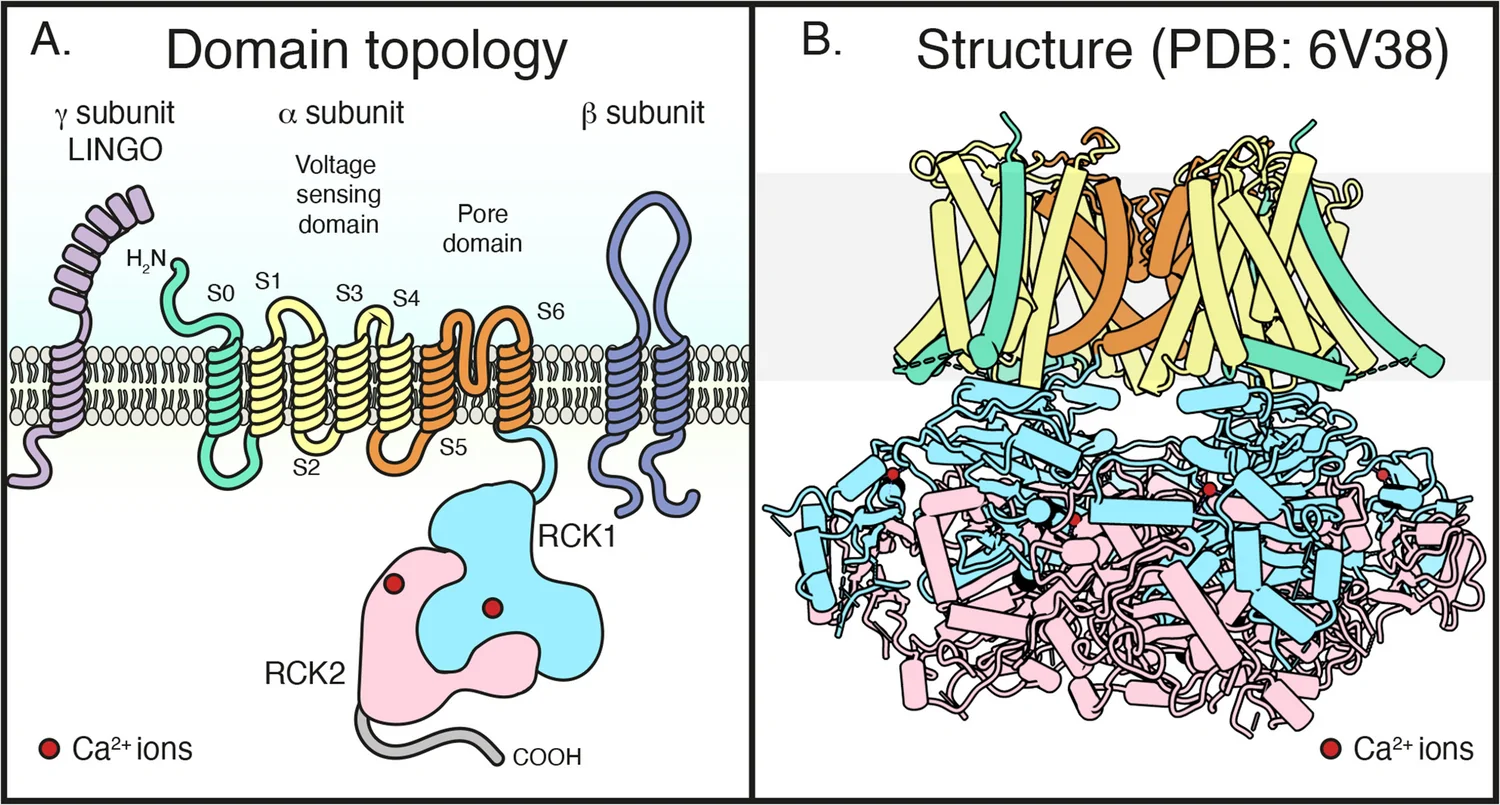
**A** Schematic domain topology of the BK channel α subunit and associated auxiliary subunits. The α subunit contains the voltage-sensing domain (S1-S4), pore domain (S5-S6), and the large cytosolic C-terminal gating ring formed by RCK1 and RCK2 domains. Auxiliary subunits γ and LINGO (left) and β subunits (right) are shown as modulatory auxiliary components. Ca^2^⁺-binding sites within the cytosolic domains are indicated. **B** Cryo-EM structure of the tetrameric BK channel (PDB: 6V38), highlighting the TM pore and voltage-sensing domains above the cytosolic gating ring. Bound Ca^2^⁺ ions are indicated

BK channels show distinctive characteristics that set them apart from Ca^2+^-gated or voltage-gated potassium channels. Guided by extensive structure–function analyses and propelled by recent advances in cryo-electron microscopy, multiple BK channel structures have been resolved in different conformational and regulatory states over the past several years (Table 2). The pore-forming BKα subunits exhibit a modular structure (Fig. 2), comprising an N-terminal membrane-embedded domain (MD) and a large cytosolic C-terminal domain (Yang and Cui 2015). The MD core shares similarities with voltage-gated potassium channels but also displays important structural differences. In BK channels, the membrane domain includes an additional transmembrane segment (S0) preceding the canonical S1–S6 segments. Accordingly, the voltage sensor domain (VSD) comprises transmembrane segments S0–S4, while the S5–S6 segments create the pore domain (PD), which serves as the ion conduction pathway (Latorre Fig. 2). Structural analyses have further clarified the organization of this voltage-sensing module (reviewed by Zhou et al. (2017); see also recent work from Contreras et al. (2025)). The extensive cytosolic C-terminal domain assembles into a characteristic “gating ring” structure (Wu et al. 2010; Yuan et al. 2011; Hite et al. 2017; Tao et al. 2017), formed by four tandems of the two highly conserved regions, the Regulator of Conductance of K^+^ (RCK1 and RCK2) domains (Giraldez and Rothberg 2017). This structural module acts as a Ca^2^⁺ sensor, with ion-binding sites distributed across the RCK1 and RCK2 domains. These sites, identified through extensive structure–function studies, enable the channel to respond to physiological Ca^2^⁺ concentrations (Schreiber and Salkoff 1997; Xia et al. 2002; Sweet and Cox 2008; Zhang et al. 2010). Within the gating ring, the BK channel’s high Ca^2+^ sensitivity has been attributed to a key binding site within RCK2, known as the “calcium bowl”, consisting of a cluster of acidic residues (Wei et al. 1994; Schreiber and Salkoff 1997; Schreiber et al. 1999). In addition, two independent Ca^2^⁺-binding sites are within the RCK1 domain: a high-affinity site in the N-terminal region and a low-affinity site in the C-terminal lobe, which is thought to be primarily occupied by Mg^2^⁺ under physiological conditions (Roosild et al. 2002; Shi et al. 2002; Xia et al. 2002; Zhang et al. 2010; Latorre et al. 2017). Together, these three sites within each RCK dimer of the gating ring (twelve sites in total) account for the channel’s Ca^2^⁺ sensitivity. Notably, replacing the C-terminal domain with that of the pH-gated SLO3 channel (Xia et al. 2004) or removing the entire gating ring (Budelli et al. 2013) eliminates all Ca^2^⁺ and Mg^2^⁺ sensitivity, confirming that Ca^2+^ binding is exclusive to the C-terminal domain.

**Table 2:** List of BK published structures, including corresponding citations and PDB references

Protein structure	Source	Identifier
Aplysia BK (Slo1) channel, Ca^2^⁺-bound	PMID: 27,974,801	PDB: 5TJI
Aplysia BK (Slo1) channel, Ca^2^⁺-bound	PMID: 27,974,795	PDB: 5TJ6
Human BK (Slo1) channel, Ca^2^⁺-bound	PMID: 31,815,672	PDB: 6V38
Human BK (Slo1) channel, Ca^2^⁺-free	PMID: 31,815,672	PDB: 6V3G
Human BK (Slo1)–β4 complex, Ca^2^⁺-bound	PMID: 31,815,672	PDB: 6V22
Human BK (Slo1)–β4 complex, Ca^2^⁺-free	PMID: 31,815,672	PDB:6V35
Drosophila BK (Slo) channel, Ca^2^⁺-free	PMID: 34,887,422	PDB:7PXF
Drosophila BK (Slo) channel, Ca^2^⁺-bound	PMID: 34,887,422	PDB:7PXE
BK (Slo1)–γ1 (LRRC26) complex	PMID: 38,035,882	PDB: 7YNZ
BK (Slo1)–γ1 (LRRC26) complex	PMID: 38,035,882	PDB: 7YO3
BK (Slo1)–γ1 (LRRC26) complex	PMID: 38,035,882	PDB: 7YO0
BK (Slo1) channel with RCK1 Ca^2^⁺-binding site mutation	PMID: 38,035,882	PDB: 7YO5
BK (Slo1) channel with RCK1 and RCK2 Ca^2^⁺-binding site mutations	PMID: 38,035,882	PDB: 7YO2
BK (Slo1)–γ1 (LRRC26) complex with RCK1 Ca^2^⁺-binding site mutation	PMID: 38,035,882	PDB: 7YO1
BK (Slo1)–γ1 (LRRC26) complex with RCK1 and RCK2 Ca^2^⁺-binding site mutations	PMID: 38,035,882	PDB: 7YO4
Rabbit BK (Slo1)–γ1 (LRRC26) complex	PMID: 38,553,946	PDB: 8S3E

**Fig. 3:**
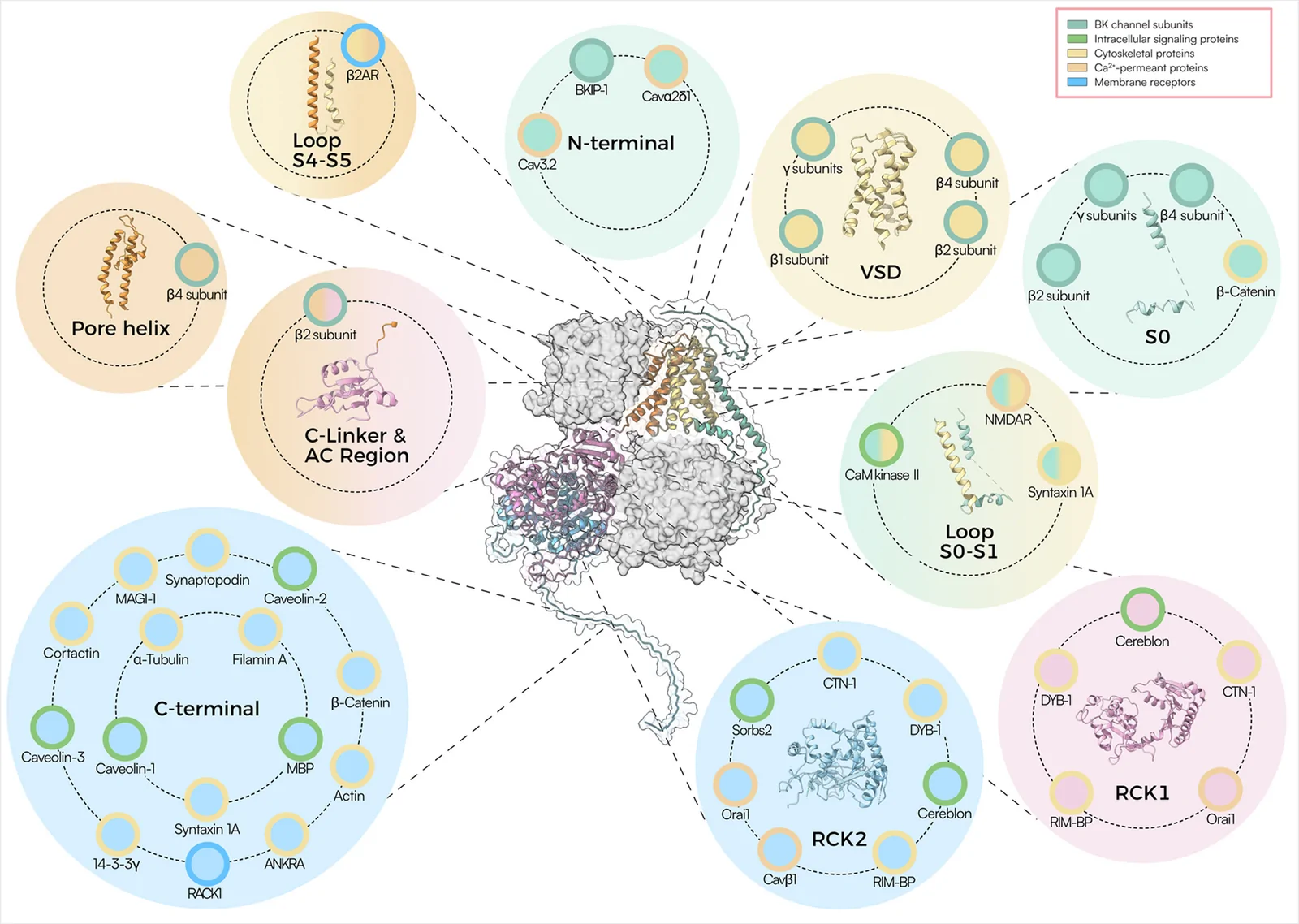
Region-specific protein–protein interactions of the BK (KCa1.1) channel. Schematic overview of proteins reported to interact with the BK channel and their mapped interaction sites. The central structure depicts the BK channel α subunit, with dashed lines linking to enlarged panels highlighting distinct channel regions, including the N-terminus, TM segments (S0, VSD, pore helix, S4–S5 loop), intracellular loops (S0–S1), C-linker/AC region, and C-terminal gating ring domains (RCK1 and RCK2). The AC region refers to the assembly/AC region near the C-linker between the pore and gating ring (often described as C-linker/AC region). Each panel lists interacting partners localized to that region (see main text for detailed information). Colored circles denote functional classes of interactors, including BK channel subunits, intracellular signaling proteins, cytoskeletal proteins, Ca^2^⁺-permeant proteins, and membrane receptors, as indicated in the legend. This figure summarizes the modular interactome of BK channels underlying their diverse regulatory mechanisms

It is through these modular regions (the TMD, VSD, and gating ring) that regulatory proteins interact with BKα tetramers, modulating channel function by altering their biophysical properties, as detailed in the following sections (Fig. 3).

## Regulatory subunits

Regulatory subunits represent the best-characterized class of BK-interacting proteins (see Table 1 for an overview). These tissue-specific subunits, which differ in structure and function, significantly influence various gating properties of BKα channels, including their kinetics, voltage dependence, apparent Ca^2^⁺ sensitivity, and pharmacological profile (for a recent review, see Dudem et al. (2021)).

Among the BK regulatory subunits, the earliest characterized are the β(1–4) accessory subunits (Garcia-Calvo et al. 1994; Wallner et al. 1999; Brenner et al. 2000; Yan and Aldrich 2010, 2012). Encoded by the genes KCNMB1-4, the β subunits have a molecular size of approximately 20 KDa and share a conserved topology consisting of two homologous TM segments (TM1 and TM2) with a large extracellular loop and intracellular short N- and C-terminal regions [Fig. 2; for extensive reviews, see (Gonzalez-Perez and Lingle 2019) and (Dudem et al. 2021)]. Each of the four β subunits exhibits distinct functional properties that align with their specific physiological roles. While they share a common mechanism to regulate BK channel function, the biophysical properties conferred by each β subunit are unique (Yang and Cui 2015). β1 and β2 enhance the apparent Ca^2^⁺ sensitivity and slow down channel gating kinetics (Latorre et al. 2017). Although β4 has a more complex regulatory role, it slows BK channel gating kinetics and alters voltage dependence, and enhancement of Ca^2^⁺ sensitivity is observed primarily at relatively high intracellular Ca^2^⁺ concentrations (> 5 μM) (Jaffe et al. 2011; Contreras et al. 2012). Unlike β2 and certain β3 splice variants, β1 and β4 do not induce inactivation or instantaneous current rectification (Dudem et al. 2021). With the exception of β3, the β1, β2, and β4 subunits have been proposed to regulate BK channel gating by stabilizing the VSD in its active conformation (Contreras et al. 2012). Additionally, the interaction between BKα and β subunits affects channel sensitivity or resistance to various pharmacological and physiological compounds (Latorre et al. 2017). For instance, BK regulation by 17β-estradiol depends on the presence of β1 or β4 (Valverde et al. 1999), which also influence the sensitivity of BK channels to alcohol (Feinberg-Zadek et al. 2008). Moreover, the β4 subunit confers resistance to iberiotoxin and charybdotoxin (Meera et al. 2000). Cryogenic electron microscopy (cryo-EM) has provided important structural insights into the interaction sites underlying regulation of BK by these auxiliary subunits (see Table 2 for specific references). It has been shown that the two TM helices of the human β4 subunits are positioned between the voltage sensor domains of the four BK subunits, while their large extracellular loop forms a crown-like structure over the central pore (Tao and MacKinnon 2019). Consistent with its known function, recent cryo-EM structure of the BKα-β2 shows the N-terminus of one of the four β subunits plugs the open pore as in ball-and-chain type inactivation (Agarwal et al. 2025). In the case of β1, and in the absence of cryo-EM structural data, biochemical approaches have led to propose complex interactions with BKα (Li and Yan 2016).

BK channel γ subunits (LRRC26/γ1, LRRC52/γ2, LRRC55/γ3, LRRC38/γ4) are single-pass leucine-rich repeat-containing proteins that dock to the BKα TM and cytosolic interface and produce large hyperpolarizing shifts in BK activation (∼15–145 mV) (Zhang and Yan 2014), thereby enabling channel opening at near-physiological voltages in many non-excitable and specialized cells (recently reviewed by Gonzalez-Perez and Lingle (2019)). Structure–function work using chimeras and mutants shows that a short region comprising the γ subunit TM helix plus the adjacent intracellular cluster of basic residues is the principal determinant of BK modulation, with different γ subunits’ TM/C-terminal juxtamembrane motifs accounting for most of their distinct gating shifts (Li et al. 2015). On the BKα side, the intracellular C-terminal region of BKα, including the highly acidic Ca^2^⁺-bowl, is indispensable for γ1 modulation and appears to form an electrostatically tuned contact zone for the positively charged γ tails, consistent with peptide mimetics of these tails shifting BK gating and with Ca^2^⁺-bowl charge neutralization weakening γ-dependent activation (Chen et al. 2023). Recent cryo-EM of the Slo1-LRRC26 complex further shows LRRC26 embracing the extracellular face of BKα to “lock” S4 voltage sensors in an active-like conformation while simultaneously stabilizing the intracellular Ca^2^⁺ sensor, illustrating dual allosteric control via extracellular contacts to S4 and intracellular contacts to the gating ring (Yamanouchi et al. 2023; Redhardt et al. 2024). However, it remains unclear whether these mechanisms are conserved across other γ subunits (Yan and Aldrich 2012; Li and Yan 2016).

LINGO proteins (LINGO1-4) constitute a distinct auxiliary subunit family, structurally related to γ subunits but with an expanded extracellular LRR/Ig domain and a short cytosolic tail (Dudem et al. 2020; Al Kawadri et al. 2025). LINGO1 and LINGO2 coassemble with BK to produce rapidly inactivating currents that activate at more negative voltages, while LINGO1 additionally suppresses BK surface expression, causing a powerful “functional knockdown” of BK activity in cells where it is expressed (Dudem et al. 2020). Functionally, LINGO1 engages BKα at multiple levels: its distal C-terminal octapeptide (RKFNMKMI) acts as an inactivation particle that likely occludes the inner pore of BKα via side portals near the S6/RCK interface and neighboring acidic residues, while its TM helix coassembles with the BKα membrane domain to stabilize the complex and shape gating (Dudem et al. 2020; Al Kawadri et al. 2025). In parallel, the large extracellular ectodomain of LINGO1 is specifically required to suppress BKα surface expression, probably through interactions with extracellular or biosynthetic trafficking determinants on BKα (Al Kawadri et al. 2025). LINGO2-containing BK channels are further tuned by redox modulation: oxidation of two methionines in the LINGO2 tail abolishes inactivation and markedly enhances BK open probability (Dudem et al. 2023). Given their largely nervous-system-enriched expression, as well as the tight association between LINGO1 upregulation, reduced cerebellar BK function, and tremor in movement disorders, LINGO subunits emerge as key determinants of cell- and disease-specific BK channel inhibition and redox sensitivity, complementing classical β and γ subunits in diversifying BK signaling (Dudem et al. 2020; Dudem et al. 2021, 2023; Al Kawadri et al. 2025). Despite growing functional evidence, structural information on the association between BKα and LINGO auxiliary proteins remains limited.

In *Drosophila*, two cytoplasmic proteins, dSLIP1 (dSlo interacting protein 1) and Slob (Slowpoke-binding protein), exemplify additional BKα-associated regulators within the BK interactome. dSLIP1 was identified by an interaction-trap screen as a PDZ-like protein that binds specifically to the C-terminal cytosolic domain of dSlo and also recognizes human BK channels; coexpression reduces macroscopic BK current primarily by decreasing channel density at the plasma membrane, consistent with a role in channel trafficking or retention (Xia et al. 1998). In contrast, Slob was discovered in a yeast two-hybrid screen as a protein that also binds the carboxy-terminal domain of dSlo, coimmunoprecipitates with dSlo from fly heads and heterologous cells, and redistributes with dSlo into shared intracellular structures (Schopperle et al. 1998). Acute application of Slob to the cytoplasmic face of patches directly increases dSlo channel activity, indicating a functional, modulatory interaction mediated via the intracellular C-terminus of BKα (Schopperle et al. 1998). Although the precise nature of the interaction remains unclear, some structural features in Slob, including proline-rich regions, a leucine zipper, and PDZ domain-like sequences, suggest potential molecular mechanisms of binding. To date, clear mammalian homologues of Slob and dSLIP1 have not been identified, suggesting that these regulators may represent invertebrate-specific BK channel accessory proteins.

In *Caenorhabditis elegans*, BKIP-1 (BK channel interacting protein) was identified genetically as a critical auxiliary subunit for the BK α subunit SLO-1 and shows direct physical association with SLO-1 via discrete interaction domains within their cytosolic regions (Chen et al. 2010) (Niu et al. 2017). BKIP-1 and SLO-1 share overlapping expression and subcellular localization, and biochemical mapping indicates specific binding rather than nonspecific membrane co-residence (Chen et al. 2010) (Niu et al. 2017). Functionally, BKIP-1 alters SLO-1 gating by slowing activation, shifting the conductance-voltage relationship in a Ca^2^⁺-dependent manner, and increasing surface expression, underscoring BKIP-1 as a *bona fide* BKα-binding regulator in the C. elegans BK interactome (Chen et al. 2010) (Niu et al. 2017).

## Cytoskeletal, scaffolding, and intracellular trafficking proteins

Among the diverse interactions involving BK channels, those with cytoskeletal, scaffolding, and intracellular trafficking proteins play a central role in regulating channel localization, membrane targeting, and turnover. These interactions contribute to the spatial organization and functional availability of BK channels at the cell surface by linking the channel to actin and microtubule networks, coordinating vesicular delivery and retrieval, and organizing channels within specialized membrane microdomains. Collectively, these studies demonstrate that interactions with cytoskeletal elements, vesicle trafficking machinery, and multidomain adaptor proteins influence BK channel surface expression, mobility, and, in some cases, gating behavior. Through these mechanisms, cytoskeletal and trafficking proteins integrate BK channels into broader cellular architectures, with important implications for their physiological roles and for channel dysregulation in pathological conditions, particularly in excitable tissues 

### Core cytoskeletal elements

In the CNS, interactions between BK channels and core cytoskeletal elements exert direct physiological effects that depend on BK channel density and activity, playing a critical role in synaptic transmission. Actin interacts directly with BK via a defined actin binding domain (ABD) in the distal C-terminus and is required for efficient trafficking of the channel to the plasma membrane, thereby supporting normal surface expression and availability (Zou et al. 2008a, b). Linkage to microtubules is mediated indirectly via MAP1A (see below) rather than direct α-tubulin binding (Ou et al. 2009). These interactions support a model in which cytoskeletal filaments provide a structural framework that facilitates BK channel delivery and stabilization at the cell surface.

### Actin binding and crosslinking proteins

Beyond core cytoskeletal filaments, several actin-binding and crosslinking proteins function as adaptors linking BK channels to the actin cytoskeleton and regulating their spatial distribution and activity. Filamin A (FLNA) and cortactin act as scaffolding proteins that couple membrane-associated complexes to actin networks. In the brain, FLNA directly binds BK channels via their cytosolic C-terminal region, primarily increasing functional current by promoting and stabilizing BK surface expression, without requiring FLNA-actin binding (Kim et al. 2007a, b). Cortactin has been shown to influence BK channel open probability and localization, consistent with a role in dynamically organizing channel positioning relative to actin structures (Park et al. 2004). Synaptopodin (Synpo), an actin-associated scaffold protein, interacts with the BK C-terminus in renal podocytes through an actin-binding domain. This interaction affects BK trafficking and membrane localization without altering channel gating properties (Kim et al. 2010). Similarly, β-catenin binds to multiple regions along the BK C-terminus, including the S10 region of the BKα subunit. This interaction promotes BK trafficking to the plasma membrane and protects the channel from proteasomal degradation, with deletion of the S10 region resulting in reduced BK surface density (Bian et al. 2011). In addition, β-catenin has been proposed to act as a physical linker between BK channels and voltage-gated Ca^2+^ channels at presynaptic terminals, implicating it in synaptic regulation (Lesage et al. 2004). Sorbs2 (Sorbin and SH3 domain-containing protein 2) represents another example of an actin-associated adaptor that directly binds both vascular BKα and BKβ subunits. The SH3 domain of Sorbs2 is required for interaction with BKα, while both SoHo and SH3 domains engage BKβ1. Structural modeling localizes Sorbs2 binding to the Ca^2^⁺ bowl region of the BKα gating ring, where it enhances BK Ca^2^⁺ sensitivity and current density (Sun et al. 2024).

### Microtubule-associated proteins

Microtubule-associated protein 1 A (MAP-1A) has been identified as an additional cytoskeletal linker that connects BK channels to the cytoskeleton. MAP-1A can associate with both microtubules and actin filaments and has been shown to influence BK channel open probability and spatial distribution in the brain, highlighting a role for microtubule-associated scaffolds in BK channel organization (Tian et al. 2006).

### Vesicle trafficking and endocytosis-exocytosis machinery

A distinct group of BK channel interactors participates in vesicle trafficking and membrane turnover. Proteins involved in endocytic mechanisms, such as dynamin-1, have been studied in the mouse brain. Gorini et al. demonstrated that BK specifically interacts with dynamin-1 but not with clathrin. This interaction was proposed to contribute to endocytic processes regulating BK channel density and assembly, as well as synaptic vesicle trafficking, release, and recycling (Gorini et al. 2010). BK channels also interact with syntaxin 1 A, a core component of the synaptic vesicle exocytosis machinery, and with its associated protein Munc18. These interactions are believed to be transient and may participate in the regulation of exocytosis (Gorini et al. 2010). Functional consequences of BK–syntaxin 1 A interactions remain debated. Cibulsky et al. reported that syntaxin 1A reduces BK channel activity, potentially by competing with Ca^2+^ channels for syntaxin binding and thereby influencing neurotransmitter release (Cibulsky et al. 2005). In contrast, earlier work by Ling et al. found that syntaxin 1A increases BK channel open probability in a Ca^2+^-dependent manner (Ling et al. 2003). These discrepancies may reflect the use of different BK channel isoforms or experimental conditions.

Control of BK intracellular trafficking has also been linked to direct interactions with the Ras-related protein Rab-4A (Rab4a), a member of the Rab family involved in endocytosis, protein trafficking, endosome fusion, and exocytosis (Wang et al. 2020) (For a recent review, see Parray (2025)).

### Membrane microdomain and caveolar scaffolds

Spatial organization of BK channel complexes at the plasma membrane is further influenced by caveolin proteins. Caveolin-1, −2, and −3 regulate the surface expression and clustering of BK channels within caveolar membrane domains, thereby shaping channel localization and signaling microenvironments (Brainard et al. 2005; Wang et al. 2005; Suzuki et al. 2013).

### Multidomain scaffolds and adaptor proteins

Several multidomain scaffolding and adaptor proteins modulate BK channel localization and activity through direct interactions. RIM-binding protein (RIM-BP) interacts directly with both voltage-gated Ca^2+^ channels and BK channels through their RCK domains. This interaction promotes trafficking of BK channels to presynaptic active zones and prevents reductions in presynaptic BK currents (Sclip et al. 2018). Analogously, in presynaptic terminals of *C. elegans*, α-catulin (CTN-1) physically interacts with the SLO-1 channel at the two RCK domains. Together with the binding of dystrobrevin (DYB-1) to the C-terminus of CTN-1, this interaction stabilizes the complex at the cell membrane and facilitates the formation of BK channel clusters near Ca^2+^ sources (Oh et al. 2015).

Ankyrin Repeat Family A protein (AnkRA) interacts with the BKα subunit in rat brain. Although this interaction does not alter BK surface expression, it modulates channel kinetics in a Ca^2+^-dependent manner, enhancing channel opening at micromolar Ca^2+^ concentrations and thereby contributing to neuronal excitability (Lim and Park 2005).

Negative regulation of BK channels can also occur through interactions with adaptor proteins. Membrane-Associated Guanylate Kinase with Inverted orientation protein 1 (MAGI-1), a member of the MAGUK family, interacts with the pore-forming domain of BK channels and reduces their membrane expression (Ridgway et al. 2009). Additionally, BK channel expression and activity can be reduced through interactions with the phospho-binding adaptor protein 14–3-3γ, acting via the ERK1/2 signaling pathway (Sokolowski et al. 2011; Chen et al. 2020).

## Ca^2+^-permeable proteins

A key feature of BK channels is their role as intracellular Ca^2+^ sensors via their RCK domains. However, BK channels exhibit relatively low affinity for Ca^2^⁺, requiring an effective concentration of approximately 10 μM Ca^2+^ for activation (Contreras et al. 2013). In many physiological contexts, BK channels are functionally coupled to Ca^2^⁺ sources, creating subcellular Ca^2^⁺ nano- or micro-domains (Lu et al. 2006; Fakler and Adelman 2008; Toro et al. 2014).

As discussed below, BK channels interact dynamically with Ca^2^⁺-permeable proteins. Voltage-gated Ca channels (VGCCs or Ca_v_) were the first identified sources functionally linked to BK activation, and they remain a prominent model for this interaction (Kim and Oh 2016). However, BK-Ca^2^⁺ source coupling extends well beyond VGCCs to encompass a broad array of other Ca^2^⁺ sources, including Store-Operated Channels (SOCs) (Wu et al. 2020), Transient Receptor Potential (TRP) channels (Wu et al. 2013; Isogai et al. 2016; Li et al. 2017), ryanodine receptors (RyRs) (Chavis et al. 1998; Irie and Trussell 2017; Harvey et al. 2020), and N-methyl-D-aspartate receptors (NMDARs) (Isaacson and Murphy 2001; Zhang et al. 2018a, b; Gomez et al. 2021). For recent reviews, see (Martinez-Lazaro et al. 2025b) and (Shah et al. 2021).

These functional associations enable BK channels to regulate a wide range of physiological processes, including smooth muscle contraction (Nelson et al. 1995; Meredith et al. 2004; Semenov et al. 2011), insulin secretion (Houamed et al. 2010), neurotransmitter release (Robitaille et al. 1993; Lingle et al. 1996; Raffaelli et al. 2004), circadian rhythm (Meredith et al. 2006; Whitt et al. 2016), action potential termination (Storm 1987; Muller et al. 2007; Montgomery and Meredith 2012), and cardiac function (Lai et al. 2014).

### Voltage-gated Ca^2+^ channels

The first functional association between VGCC and BK channels was demonstrated in the late 1990 s (Marrion and Tavalin 1998; Prakriya and Lingle 1999), and subsequent studies established the concept of BK channels forming signaling complexes with Ca^2^⁺ sources that enable rapid and localized Ca^2^⁺-dependent activation (Lu et al. 2006; Toro et al. 2014). This was later supported by key biochemical and molecular evidence (Grunnet and Kaufmann 2004; Berkefeld et al. 2006). Together, these and subsequent studies have identified several VGCC subtypes that physically and functionally associate with BK channels: L-type (Ca_v_1.2, Ca_v_1.3) (Grunnet and Kaufmann 2004; Berkefeld et al. 2006; Berkefeld and Fakler 2008; Marcantoni et al. 2010; Vivas et al. 2017; Whitt et al. 2018; McNally et al. 2021), P/Q type (Ca_v_2.1) (Berkefeld et al. 2006; Berkefeld and Fakler 2008), N-type (Ca_v_2.2) (Berkefeld et al. 2006; Loane et al. 2007), and T-type (Ca_v_3.2) (Rehak et al. 2013). Although R-type Ca^2+^ channels (Ca_v_2.3) were initially thought not to form complexes with BK channels (Berkefeld et al. 2006; Cerrada et al. 2018), recent evidence suggests that such interactions can occur, at least in specific cellular contexts (Gutzmann et al. 2019).

The functional effects of BK channels coupling to specific Ca_v_ subtypes may vary with cellular localization, shaping neuronal physiology in a context-dependent manner. For example, Ca_v_1.3 has been shown to form nanodomain clusters with BK in sympathetic and hippocampal neurons, allowing BK activation at more negative voltages (as low as −50 mV), which is critical for regulating excitability near action potential threshold (Vivas et al. 2017). Similarly, the Ca_v_3-BK interaction was found to activate BK near −50 mV to contribute to spike repolarization and firing gain (Rehak et al. 2013). In contrast, Ca_v_1.2 shifts BK activation to more depolarized potentials compared to Ca_v_1.3 (Berkefeld and Fakler 2008). P/Q- and N-type Ca^2+^ channels rapidly and effectively activate BK channels, with Ca_v_2.1 showing the most abundant association in brain tissue (Loane et al. 2007). High-resolution imaging further corroborated that BK and Ca_v_2.1 clusters colocalize in cerebellar Purkinje cell soma and dendrites (Indriati et al. 2013). In CA1 pyramidal cells, coupling of Ca_v_2.3 to BK results in changes in AP waveform and synaptic plasticity (Gutzmann et al. 2019).

Further studies have explored the domains mediating VGCC-BK channel associations, although for most subtypes, the specific regions involved remain undefined. It has been proposed that Ca_v_1.2, Ca_v_2.1, Ca_v_2.2, and Ca_v_2.3 physically interact with BK channels via their α subunits (Berkefeld et al. 2006; Gutzmann et al. 2019). Some evidence suggests that Ca_v_1.3 channels may couple to BK through the auxiliary BKβ2 subunit (Marcantoni et al. 2010). Additionally, Ca_v_3.2 has been proposed to interact with BK channels via the S0 TM segment in the N-terminal region, extending the voltage range of BK activation (Rehak et al. 2013). Auxiliary subunits of Ca_v_ channels have also been shown to interact with BK channels, leading to distinct functional effects. The Ca_v_α2δ1 subunit forms a high-affinity interaction with the N-terminal region of BK, resulting in reduced Ca_v_2 membrane abundance (Zhang et al. 2018a, b). Meanwhile, the Ca_v_β1 subunit slows BK channel activation by interacting with the calcium bowl region, reducing Ca^2+^ sensitivity without affecting the channel’s relative membrane expression (Zou et al. 2008a, b).

### Non-voltage-gated Ca^2+^-permeable channels and receptors

Non-voltage-gated Ca^2^⁺ channels are crucial for diverse cell functions beyond electrical signaling, including families like the store-operated channels (SOCs), primarily involving proteins Orai and STIM; ryanodine receptors (RyRs); IP_3_ receptors (IP_3_Rs); N-methyl-D-aspartate receptors (NMDARs); as well as members of the transient receptor potential (TRP) family. Associations of BK have been reported with most of these channels, forming specialized signaling complexes that shape essential physiological functions like gene expression, secretion, cell proliferation, and calcium homeostasis.

SOCs are Ca^2+^-selective channels mediating the store-operated calcium entry (SOCE), an essential pathway for cell calcium homeostasis (Putney 1986; Prakriya and Lewis 2015). The principal components of SOCs are proteins STIM (stromal interaction molecule) and Orai, each comprising several homologs (Prakriya and Lewis 2015). BK channels have been shown to functionally couple to SOCs, particularly with Orai1, as confirmed by immunoprecipitation and co-localization studies (Chen et al. 2016). This interaction appears to involve regions within BK’s RCK1 (amino acids 350 to 371) and RCK2 (amino acids 720 to 814) domains (Wu et al. 2020). In rat mesenteric artery smooth muscle, Ca^2+^ influx through SOCs activates BK channels, leading to membrane hyperpolarization that enhances the driving force for further Ca^2+^ entry via SOCs, therefore establishing a positive feedback loop that amplifies calcium signaling (Chen et al. 2016).

There is strong evidence for both functional and physical interactions between BK channels at the plasma membrane and intracellular Ca^2+^ release channels, IP_3_R and RyR, across several cell types, especially in smooth muscle and neurons. In arterial smooth muscle cells, IP_3_R1 activation at specific locations of the endoplasmic reticulum (ER) has been shown to increase the apparent Ca^2+^ sensitivity of plasma membrane BK channels through localized molecular coupling, establishing a negative feedback mechanism that limits IP_3_-induced vasoconstriction in cerebral arteries (Zhao et al. 2010; Yang et al. 2013). Interestingly, in urinary bladder smooth muscle cells, IP_3_R-BK associations have also been proposed to contribute to IP_3_-mediated contractility; however, the relative involvement of BK versus TRPM4 in this process remains under debate (Parajuli et al. 2016). Co-localization and functional association between intracellular IP_3_R and BK channels at the neuronal plasma membrane have additionally been described in glioma cells (Weaver et al. 2007) and in Purkinje neurons. In the latter, they are proposed to form part of a functional unit termed the *plasmerosome* involved in the regulation of local Ca^2+^ signaling and neuronal function (Kaufmann et al. 2009).

In smooth muscle, transient release of Ca^2+^ from the ER, mediated by activation of RyRs, activates nearby BK channels, inducing membrane hyperpolarization and thereby modulating vascular muscle tone (Nelson et al. 1995; Bolton and Imaizumi 1996; Perez et al. 1999; Herrera et al. 2001). In interneurons of the dorsal cochlear nucleus, RyRs have been reported to form triad couplings, where both plasma membrane BK and Ca_v_ channels couple to RyRs in the ER, providing a mechanism for the rapid control of action potentials (Irie and Trussell 2017). A distinct configuration has been described in cerebral artery smooth muscle cells, where functional associations among BK, RyRs, and TRPV4 underlie arterial dilation in response to an endothelial-derived factor (Earley et al. 2005). Despite evidence for functional coupling, direct physical interactions between RyR and BK channels have not been conclusively demonstrated by biochemical approaches such as co-immunoprecipitation. Recent studies suggest that any physical interaction of BK and RyR may be indirect and mediated by scaffolding proteins, including junctophilin-2 and Caveolin-1 (Pritchard et al. 2019; Saeki et al. 2019).

Early studies in hippocampal neurons provided the first evidence that Ca^2^⁺ influx through NMDARs can activate nearby Ca^2^⁺-activated K⁺ channels (Nicoll and Alger 1981; Zorumski et al. 1989), a mechanism later clearly demonstrated in the olfactory bulb where NMDAR-mediated Ca^2^⁺ entry activates BK channels (Isaacson and Murphy 2001). Subsequent work established that BK and NMDARs form functional and biochemical complexes across multiple brain regions, including hippocampus, cortex, cerebellum, striatum, thalamus, nucleus accumbens, dorsal cochlear nucleus, and spinal cord (He et al. 2014; Ji et al. 2015; Zhang et al. 2018a, b; Fan et al. 2021). These complexes have been shown to operate at both dendritic and somatic compartments, as well as at synaptic and extrasynaptic sites, where NMDAR-derived Ca^2^⁺ activates BK to shape neuronal excitability and synaptic responses (Isaacson and Murphy 2001; Zhang et al. 2018a, b; Gomez et al. 2021; Tazerart et al. 2022; Mitchell et al. 2023; Reyes-Carrión et al. 2023). Functional coupling has also been recapitulated in heterologous expression systems, supporting the existence of NMDAR-BK nanodomains and enabling mechanistic dissection of subunit composition, mutations, and coupling specificity (Zhang et al. 2018a, b; Gomez et al. 2021; Martínez-Lázaro et al. 2025b). A recent comprehensive review provides a more detailed discussion of this topic (Martinez-Lazaro et al. 2025a).

Transient receptor potential (TRP) channels are a diverse family of non-selective cation channels that mediate Ca^2^⁺ influx in response to various chemical and physical stimuli (Clapham 2003; Vangeel and Voets 2019). Several members of the TRP family converge functionally on BK channels across a wide range of tissues, positioning BK as an integrative node within Ca^2^⁺-dependent signaling networks. In vascular smooth muscle cells, Ca^2^⁺ influx through TRPC1 has been shown to activate BK channels, leading to membrane hyperpolarization and preventing excessive agonist-induced contraction (Kwan et al. 2009). In kidney podocytes, TRPC3 and TRPC6 physically interact with BK channels, contributing to larger signaling assemblies that regulate foot process architecture and kidney function (Kim et al. 2009). In this physiological context, TRPC6 has been shown to increase the surface expression of the BKα subunit, whereas TRPC3 does not (Kim et al. 2009). In dorsal root ganglion (DRG) neurons, a BK-mediated negative feedback mechanism involving TRPV1-BK macrocomplexes has been proposed to modulate pain perception (Wu et al. 2013). Although physical colocalization between BK and TRPV4 has not yet been demonstrated, functional coupling has been described in different physiological settings, including human bronchial epithelial cell lines (Fernandez-Fernandez et al. 2008). In cerebral arterial smooth muscle cells, TRPV4 has been proposed to activate BK channels via RyR-mediated Ca^2^⁺ release to induce arterial relaxation (Earley et al. 2005). Under pathological conditions following traumatic brain injury, mitochondrial-derived H₂O₂ activates TRPV4, leading to enhanced BK channel activity and impaired myogenic constriction, independent of RyR-mediated Ca^2^⁺ signaling (Szarka et al. 2018). Finally, a functional interaction between BK and TRPM8 has been proposed to regulate menthol-stimulated cell migration in human glioblastoma cells (Wondergem and Bartley 2009).

## Signalling proteins

BK channels participate in an extensive interactome composed of membrane receptors and intracellular signaling molecules that, together with scaffolding proteins, shape channel localization and functional output. Within this network, membrane-associated proteins such as the receptor for activated C kinase 1 (RACK1) interact with the C-terminal region of the BK channel, forming part of a regulatory complex that measurably modifies BK gating properties, likely by scaffolding kinases and other signaling partners onto the channel (Isacson et al. 2007; Surguchev et al. 2012). Similarly, β_2_-adrenergic receptors (β_2_AR) form macromolecular complexes with BK channels and directly scaffold their regulation (Chanrachakul et al. 2004; Liu et al. 2004).

A membrane-delimited signaling mechanism has also been described in which G protein-coupled receptors (GPCRs) locally regulate BK channels through the release of Gβγ subunits that bind directly to the channel. Muscarinic M2 receptors (M2R) inhibit BK channels by liberating Gβγ, which directly associates with BK, while simultaneously engaging a Gβγ-PLC-PKC signaling cascade that provides an additional indirect inhibitory component (Guo et al. 2012). In contrast, activation of melatonin MT1 receptors (MT1R) also signals through Gβγ but results in enhanced BK channel activity (Niu et al. 2020). Together, these findings support a unified model in which distinct GPCRs modulate BK channel gating and assembly via direct interactions with G protein subunits, analogous to the regulation of other ion channels such as GIRK and Kv channels.

There is strong evidence that intracellular kinases regulate BK channels through phosphorylation of defined C-terminal sites. Although phosphorylation does not necessarily imply stable physical association, kinase-mediated regulation functionally integrates BK channels into broader signaling complexes that form a dynamic component of the BK interactome. cGMP-dependent protein kinase (PKG) (Fukao et al. 1999; White et al. 2000; Dong et al. 2008; Bollinger et al. 2018), protein kinase A (PKA) (Reinhart et al. 1991), and protein kinase C (PKC) (van Welie and du Lac 2011) phosphorylate the BKα subunit at distinct residues. In neurons and other excitable cells, BK channels are also subject to Ca^2^⁺/calmodulin-dependent phosphorylation by CaMKII, resulting in transient modulation of channel activity (Liu et al. 2006). Finally, overexpression of the glycogen synthase kinase 3 β (GSK3β) in Xenopus oocytes was shown to up-regulate BK activity, with a proposed role in cell volume and excitability (Sokolowski et al. 2011; Fezai et al. 2016). These phosphorylation events highlight the integration of BK channels into dynamic signaling networks, although many of these regulatory mechanisms likely involve transient enzymatic interactions rather than stable channel-associated complexes. A comprehensive discussion of kinase and phosphatase signaling pathways is beyond the scope of this focused review.

Consistent with the interactome framework adopted in this review, serine/threonine kinases predominantly regulate BK channels through phosphorylation-dependent mechanisms that do not require stable physical association, whereas several tyrosine kinases engage BK through more direct protein–protein interactions that integrate the channel into signaling assemblies. In several mammalian systems, phosphorylation by the proto-oncogene tyrosine-protein kinase Src produces functionally important increases in BK current and Ca^2^⁺ sensitivity, particularly in the context of integrin-linked signaling (Wang et al. 1999; Ling et al. 2004). In addition, current evidence supports that the active proline-rich tyrosine kinase 2 (PyK2) co-associates with BK channels and enhances Ca^2^⁺-dependent gating via tyrosine phosphorylation of the BKα subunit, although an unambiguously direct, adaptor-free BK–PyK2 binary interaction has not been demonstrated (Ling et al. 2004). Finally, the non-receptor tyrosine kinase Syk binds directly to the BK channel through a Src-phosphorylated ITAM motif (Tyr1061/Tyr1072) within the hSlo C terminus, forming a signaling platform (Rezzonico et al. 2002). Notably, recruitment of Syk does not measurably alter BK gating, suggesting a scaffold or signal-transduction role rather than direct modulation of channel activity (Rezzonico et al. 2002).

Additional proteins acting as signal-responsive regulatory hubs expand the functional diversity of the BK interactome. One example is Heme oxygenase-2 (HO-2), an oxygen sensor that increases channel activity in normoxia by binding to the cytosolic heme-binding domain (HBD) in the RCK1-RCK2 linker of BKα (Williams et al. 2004; Yi et al. 2010). Functionally, the HO-2/BK complex enables redox- and O_2_-dependent regulation of BK activity via local control of heme, CO, and Fe^2^⁺ near the HBD, with the CXXCH motif in the HBD acting as a thiol/disulfide redox switch that tunes both heme/CO affinity and HO-2/HBD association (Yi et al. 2010). Another example is Cereblon (CRBN), a signal-regulated E3-ligase adapter that controls multiple canonical signaling pathways via ubiquitin-mediated processing of key signaling proteins, which has been identified as a binding partner of BK channels (Jo et al. 2005). Functionally, CRBN acts mainly as a negative regulator of BK surface expression and current. Disruption of this interaction—either increasing or decreasing BK activity depending on the mutation or ligase pathway involved—alters presynaptic release and cognitive function (Choi et al. 2018).

Finally, although direct physical interactions have not been experimentally demonstrated, proteins involved in post-translational regulation also shape BK interactome dynamics. Although this review does not aim to cover all such regulators, it is worth noting that several act on BK channels to exert their effects. For example, the α/β-hydrolase domain-containing protein 17a (ABHD17a) and the acyl protein thioesterase Lypla1 regulate deacylation of BK channel domains, thereby influencing channel activity and membrane retention (McClafferty et al. 2020) (for a review on this topic, see, e.g., Shipston and Tian (2016)).

Together, these interactions position BK channels as central hubs within multiprotein signaling assemblies, enabling precise, context-dependent regulation of cellular processes including neurotransmission, smooth muscle contractility, and cellular stress responses.

## Other interacting proteins

This category includes a diverse set of proteins with important roles in the nervous system that do not fit neatly into receptors, signaling molecules, or enzymatic regulators. These include transporters (GAT3), RNA-binding translational regulators (FMRP), axonal adhesion and scaffolding proteins (Caspr), structural myelin proteins (MBP), metabolic or chaperone proteins (ApoA1, HSP60), as well as an ion channel (KCa3.1). Although heterogeneous, these interactions highlight additional mechanisms through which BK channels are integrated into neuronal and glial function.

Fragile X mental retardation protein (FMRP), an RNA-binding translational regulator, also acts as a direct protein partner of BK channels. FMRP binds the auxiliary β4 subunit and enhances BK Ca^2^⁺ sensitivity, thereby constraining presynaptic action potential broadening, Ca^2^⁺ influx, and neurotransmitter release in hippocampal and cortical pyramidal neurons (Deng et al. 2013; Deng and Klyachko 2016; Kshatri et al. 2020). Beyond this β4-mediated mechanism, FMRP directly modulates BKα- and BKαβ4-containing channels, slowing gating kinetics and favoring channel opening. Loss of FMRP or disease-linked FMRP mutants (e.g., R138Q or BK-binding-deficient variants) reduces BK open probability and contributes to hyperexcitability and fragile X syndrome-like synaptic and circuit defects, which can be partially rescued by genetic or pharmacological upregulation of BK activity (Deng and Klyachko 2016; Kshatri et al. 2020).

Apolipoprotein A1 (ApoA1), the major structural protein of high-density lipoproteins (HDL), directly interacts with BK channels in cochlear tissue (Sokolowski et al. 2009). ApoA1 binding inhibits BK channel activity by slowing gating kinetics and shifting the voltage dependence of activation toward more positive potentials, thereby reducing channel voltage sensitivity (Sokolowski et al. 2009). This interaction links lipid-handling proteins to BK channel regulation and suggests a potential connection between ApoA1 biology and neuronal excitability.

The intermediate-conductance Ca^2^⁺-activated K⁺ channel KCa3.1 (also known as IK1) provides a well-defined example of direct protein–protein inhibition of BK channels. Activation of KCa3.1 suppresses BK activity even in excised membrane patches, and an N-terminal KCa3.1 peptide blocks BK by occluding the cytoplasmic pore with minimal effects on BK gating kinetics. This interaction shapes Ca^2^⁺-activated K⁺ flux in secretory epithelia (Thompson and Begenisich 2006, 2009).

Proteomic analyses of mouse brain tissue have identified the GABA transporter 3 (GAT3) as a previously unrecognized binding partner of the BKα-subunit. GAT3 is physically associated with BK channels; however, the precise molecular mechanism by which GAT3 modulates BK gating remains undefined. Given that both BK channels and GAT3 are regulated by intracellular Ca^2^⁺, their interaction has been proposed to form a functional microdomain in which BK activity influences GABA uptake and release by astrocytes (Singh et al. 2016).

Finally, myelin basic protein (MBP) was identified in a proteomics-based study as a direct interactor of the BKα C-terminal region. MBP binding shifts BK conductance-voltage activation curves toward more negative potentials and also interacts with calmodulin (CaM), suggesting a potential role in linking BK channels to CaM-dependent signaling pathways (Kim et al. 2007a, b).

## Conclusions and future directions

BK channels occupy a unique position at the interface between membrane excitability and intracellular Ca^2^⁺ signaling. Beyond their intrinsic biophysical properties, it is increasingly clear that the extraordinary functional versatility of BK channels arises from their participation in highly organized, multiprotein signaling assemblies. As highlighted in this review, BK channels interact with a remarkably diverse set of partners, including regulatory subunits, cytoskeletal and trafficking proteins, Ca^2^⁺-permeable channels and receptors, and intracellular signaling molecules (Fig. 1 and Table 1). Where evidence supports direct physical association or complex formation, these interactions provide molecular mechanisms for tuning channel gating, surface expression, subcellular localization, and coupling to local Ca^2^⁺ sources, enabling BK channels to operate over a wide range of temporal and spatial scales in both excitable and non-excitable cells.

A recurring theme emerging from the BK interactome is its strong context dependence. The functional outcome of BK channel activity is dictated not only by channel isoform or auxiliary subunit composition, but also by the specific molecular environment in which the channel is embedded. Distinct nano- and microdomain configurations, such as those formed with voltage-gated Ca^2^⁺ channels, TRP channels, and neurotransmitter receptors, and through functional coupling to intracellular Ca^2^⁺ release pathways, enable BK channels to decode local Ca^2^⁺ signals and translate them into precise changes in membrane potential and cellular output. In addition to plasma membrane signaling complexes, BK channel assemblies have also been reported in other subcellular compartments, including mitochondria (Singh et al. 2013), lysosomes (Cao et al. 2015), and extracellular vesicles (Sanghvi et al. 2025), suggesting that the composition and functional consequences of BK channel complexes may vary substantially depending on cellular localization and cell type. For example, mitochondrial BK channels have been proposed to interact with components of the mitochondrial import machinery such as TOM22 (Zhang et al. 2017). Similarly, interactions with scaffolding proteins, cytoskeletal elements, and signaling enzymes confer dynamic regulation of BK channel trafficking, clustering, and post-translational modification, linking BK function to broader cellular signaling networks. The growing set of additional BK-associated proteins with specialized neuronal and glial roles further suggests that this interactome extends beyond canonical channel regulators.

Despite substantial progress, important questions remain. For many reported interactions, the precise molecular interfaces, stoichiometries, and structural arrangements are still unknown, and it is often unclear whether associations are direct or mediated by additional scaffolding components. Advances in cryo-EM, super-resolution imaging, and proximity-based proteomics are likely to be pivotal in resolving these issues. Moreover, understanding how disease-associated mutations in BK channels or their interacting partners perturb these complexes will be essential for linking BK channel dysfunction to neurological, cardiovascular, and metabolic disorders. Ultimately, a more integrated view of BK channels as dynamic hubs within specialised signalling assemblies may open new avenues for therapeutic intervention aimed at selectively targeting pathological BK channel signalling without disrupting their essential physiological roles.

## Data Availability

All information related to this article can be provided by the authors upon reasonable request.
